# Peroxisomal Localization of Benzyl Alcohol *O*-Benzoyltransferase HSR201 is Mediated by a Non-canonical Peroxisomal Targeting Signal and Required for Salicylic Acid Biosynthesis

**DOI:** 10.1093/pcp/pcae129

**Published:** 2024-10-29

**Authors:** Yu Kotera, Yoshika Asai, Shutaro Okano, Yukako Tokutake, Akira Hosomi, Katsuharu Saito, Shinichi Yonekura, Shinpei Katou

**Affiliations:** Graduate School of Science and Technology, Shinshu University, Minamiminowa 8304, Nagano 399-4598, Japan; Graduate School of Science and Technology, Shinshu University, Minamiminowa 8304, Nagano 399-4598, Japan; Graduate School of Science and Technology, Shinshu University, Minamiminowa 8304, Nagano 399-4598, Japan; Graduate School of Science and Technology, Shinshu University, Minamiminowa 8304, Nagano 399-4598, Japan; Graduate School of Science and Technology, Shinshu University, Minamiminowa 8304, Nagano 399-4598, Japan; Graduate School of Science and Technology, Shinshu University, Minamiminowa 8304, Nagano 399-4598, Japan; Graduate School of Science and Technology, Shinshu University, Minamiminowa 8304, Nagano 399-4598, Japan; Graduate School of Science and Technology, Shinshu University, Minamiminowa 8304, Nagano 399-4598, Japan

**Keywords:** Disease resistance, Hypersensitivity-related genes, *Nicotiana benthamiana*, *Nicotiana tabacum*, Peroxisomal β-oxidation, Salicylic acid

## Abstract

The phytohormone salicylic acid (SA) regulates plant responses to various types of environmental stress, particularly pathogen infections. We previously revealed that the benzyl alcohol *O*-benzoyltransferase HSR201 was required for pathogen signal-induced SA synthesis, and its overexpression together with *NtCNL*, encoding a cinnamate-coenzyme A ligase, was sufficient for the production of significant amounts of SA in tobacco. We herein examined the subcellular localization of HSR201 and found that it fused to a yellow fluorescent protein localized in peroxisomes. Most peroxisomal matrix proteins possess peroxisomal targeting signal type-1 (PTS1) located at the extreme C-terminus or PTS2 located at the N-terminus; however, a bioinformatics analysis failed to identify similar signals for HSR201. Deletion and mutation analyses of HSR201 identified one essential (extreme C-terminal Leu^460^) and three important (Ile^455^, Ile^456^ and Ala^459^) amino acid residues for its peroxisomal localization. The virus-induced gene silencing (VIGS) of *PEX5*, a PTS1 receptor, but not *PEX7*, a PTS2 receptor, compromised the peroxisomal targeting of HSR201 in *Nicotiana benthamiana*. When overexpressed with NtCNL, HSR201 mutants with reduced or non-peroxisomal targeting induced lower SA levels than the wild type; however, these mutations did not affect the protein stability or activity of HSR201. VIGS of the *HSR201* homolog compromised pathogen signal-induced SA accumulation in *N. benthamiana*, which was complemented by the HSR201 wild type, but not the mutant with non-peroxisomal targeting. These results suggest that the peroxisomal localization of HSR201 is mediated by a non-canonical PTS1 and required for SA biosynthesis.

## Introduction

Peroxisomes are small single membrane-bound organelles that are present in nearly all eukaryotes. In plants, peroxisomes contribute to a wide range of physiological functions, such as lipid metabolism, the generation and detoxification of reactive oxygen species, photorespiration and the biosynthesis of phytohormones (Kao et al. 2018). In lipid metabolism, fatty acids are catabolized into acetyl-coenzyme A (CoA) by fatty acid β-oxidation, which is required for seed germination and post-germinative growth. Fatty acid β-oxidation occurs through multiple enzymes: fatty acid CoA ligase, acyl-CoA oxidase (ACX), multifunctional protein (MFP) and 3-ketoacyl-CoA thiolase (KAT). Fatty acid CoA ligases produce acyl-CoA by attaching CoA to fatty acids. ACXs oxidize acyl-CoA to *trans*-enoyl-CoA. MFPs exhibit hydratase and dehydrogenase activities and convert *trans*-enoyl-CoA to 3-ketoacy-CoA. KATs cleave-off acetyl-CoA, which shortens the original acyl-CoA by a C_2_ unit.

Beyond energy production, β-oxidation in peroxisomes contributes to the biosynthesis of phytohormones (Kao et al. 2018). Jasmonic acid (JA) is a phytohormone that regulates reproductive development and responses to wounds and biotic stress. It is produced from its precursor 12-oxo-phytodienoic acid by a reduction and three cycles of β-oxidation in peroxisomes (Wasternack and Song 2017). The conversion of indole-3-butyric acid (IBA), an auxin precursor, to indole-3-acetic acid, the main auxin in plants, is also catalyzed by β-oxidation in peroxisomes; however, some of the enzymes involved in the β-oxidation of IBA differ from those that play a role in fatty acid β-oxidation (Frick and Strader 2018). IBA and IBA-derived auxin are essential in various aspects of root and shoot development. The biosynthesis of the phytohormone salicylic acid (SA) is also mediated by peroxisomal β-oxidation. SA regulates plant responses to various forms of environmental stress, particularly pathogen infections, and is referred to as a defense hormone (Peng et al. 2021). Similar to auxin, SA is produced through at least two different pathways, the isochorismate synthase (ICS) and phenylalanine ammonia lyase (PAL) pathways, with peroxisomal β-oxidation contributing to the latter pathway. PAL is the first enzyme of the general phenylpropanoid pathway and converts phenylalanine to *trans*-cinnamic acid (CA). Although the molecular mechanisms underlying the PAL pathway remain unclear, previous studies suggested the conversion of CA to SA in part by peroxisomal β-oxidation. In *Arabidopsis thaliana*, stress-induced SA synthesis in leaves predominantly occurs through the ICS pathway; however, peroxisomal β-oxidation is required for SA production during seed development (Peng et al. 2021). SA levels were previously shown to be slightly and markedly reduced in seeds with a defect in *BZO*1 encoding CoA ligase and *ABNORMAL INFLORESCENCE MERISTEM 1* (*AIM1*), one of the two genes for MFP, respectively (Bussell et al. 2014). Furthermore, rice (*Oryza sativa*) was found to contain high basal levels of SA, which required an *AIM1* homolog (Xu et al. 2017, 2023). Although the precise roles of *Arabidopsis* and rice AIM1 in SA biosynthesis are unclear, they are similar to petunia cinnamoyl-CoA hydratase/dehydrogenase PhCHD (Qualley et al. 2012), and probably contribute to the conversion of CA to benzoic acid.

**Fig. 1:**
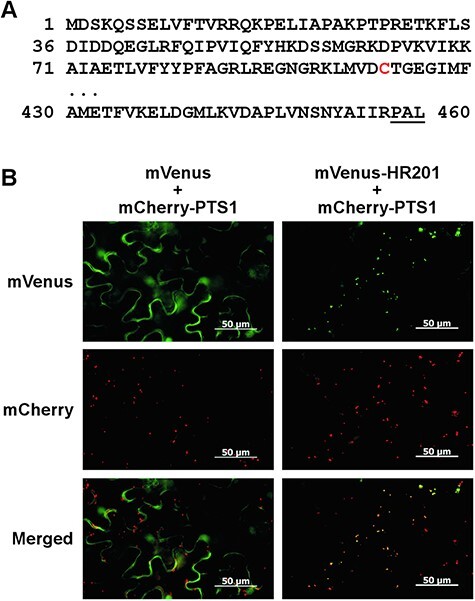
HSR201 mainly localizes in peroxisomes via a non-canonical PTS. (A) Amino acid sequences of the N- and C-terminal regions of HSR201. The first Cys residue in the N-terminal region is shown in red. The extreme C-terminal tripeptide is underlined. (B) HSR201 mainly localizes in peroxisomes. *Agrobacterium* cells carrying mVenus-HSR201 expressed from a modified 35S promoter were mixed with those containing mCherry-PTS1 expressed from the same promoter at a ratio of 1: 1, and the mix was then infiltrated into *N. benthamiana* leaves. As a control, mVenus alone was expressed in a similar manner. Two days after infiltration, fluorescence was observed. Bar = 50 μm.

We recently revealed that the hypersensitivity-related protein HSR201 and the peroxisomal β-oxidative pathway consisting of NtCNL, NtCHD and NtKAT1 cooperatively contributed to SA biosynthesis in tobacco (*Nicotiana tabacum* and *N. benthamiana*; Takagi et al. 2022, Kotera et al. 2023). HSR201 is a benzyl alcohol *O*-benzoyltransferase (BEBT) that catalyzes the conjugation of benzoyl-CoA (BA-CoA) to benzyl alcohol, resulting in the formation of benzyl benzoate (D’Auria et al. 2002). NtCNL, NtCHD and NtKAT1 are homologous to cinnamate-CoA ligase, cinnamoyl-CoA hydratase/dehydrogenase and KAT, respectively, and their recombinant proteins converted CA to benzoyl-CoA, a preferred substrate of HSR201, suggesting that they function between PAL and HSR201 in SA biosynthesis (Kotera et al. 2023). A subcellular localization analysis confirmed that NtCNL, NtCHD and NtKAT1 mainly localized in peroxisomes (Kotera et al. 2023). The majority of peroxisomal matrix proteins possess peroxisomal targeting signal type-1 (PTS1) or PTS2. PTS1 is a tripeptide located at the extreme C terminus of proteins with a consensus sequence of [S/A]-[K/R]-[L/M/I], and the majority of peroxisomal matrix proteins contain PTS1 (Reumann et al. 2016). Although diverse non-canonical amino acid residues are present in PTS1 sequences, almost all experimentally verified PTS1 sequences contain canonical amino acid residues at least at two of the three positions (Reumann et al. 2016, Deng et al. 2022). PTS2 is a cleavable N-terminal presequence with a consensus sequence of [RK]-[LVIQ]-[RK]-[LVIQ]-[LVIHQ]-[LSGAK]-X-[HQ]-[LAF]) (where X indicates any amino acid), and a smaller number of peroxisomal matrix proteins harbor PTS2 (Petriv et al. 2004). After delivery into peroxisomes, the N-terminal presequence is cleaved at the conserved Cys-containing motif closely downstream of the consensus sequence. NtCNL and NtCHD have the canonical PTS1 sequences SRL and SRI, respectively, whereas NtKAT1 possess the canonical PTS2 sequence RQRVLLEHL (Kotera et al. 2023). On the other hand, HSR201 has no PTSs (**Fig. 1A**) and protein-targeting prediction programs predicted no specific subcellular localization for HSR201; however, we found that it mainly localized in peroxisomes. In the present study, we investigated the molecular mechanisms underlying the peroxisomal localization of HSR201, and the results obtained showed that it was mediated by a non-canonical PTS1 and required for SA biosynthesis.

## Results

### HSR201 mainly localizes in peroxisomes

To examine the subcellular localization of HSR201, it was fused to the C-terminus of mVenus (mVenus-HSR201), an enhanced yellow fluorescent protein (Kremers et al. 2006). As a peroxisome marker, an enhanced red fluorescent protein (Shaner et al. 2004) containing Ser-Lys-Leu, a typical PTS1 sequence, at its C-terminus (mCherry-PTS1) was used. They were expressed under the control of a strong constitutively active promoter in *N. benthamiana* leaves using *Agrobacterium*-mediated transformation. As shown in **Fig. 1B**, mVenus-HSR201 and mCherry-PTS1 showed similar punctate fluorescence patterns, most of which overlapped with each other. On the other hand, fluorescence from mVenus alone was mainly observed in the periphery of cells and did not overlap with fluorescence from mCherry-PTS1. These results indicate that HSR201 mainly localized in peroxisomes.

### Identification of a non-canonical PTS of HSR201

**Fig. 2:**
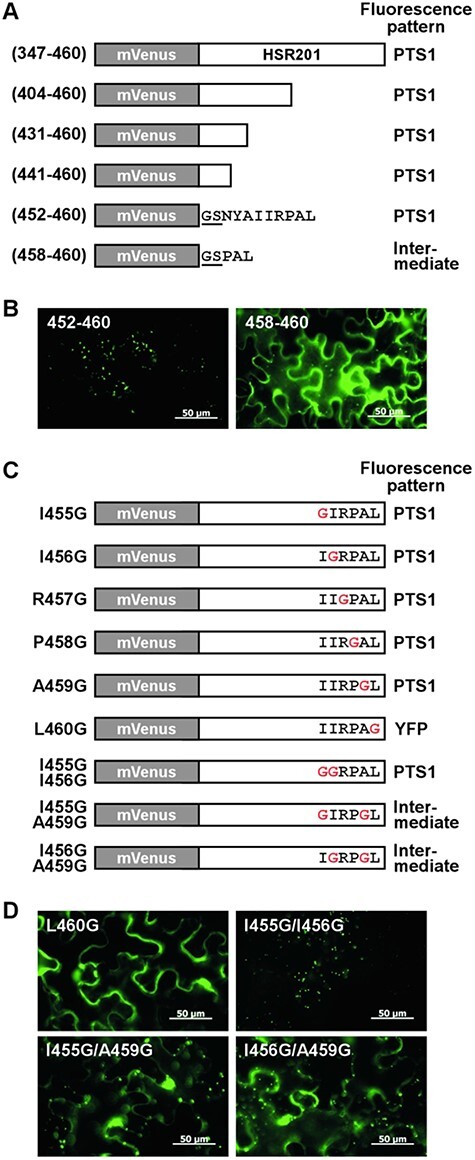
Identification of amino acid residues of HSR201 required for its peroxisomal localization. (A) A schematic representation of the deletion constructs of HSR201 fused to mVenus. The Gly-Ser residues resulting from the *Bam*HI site used to fuse the gene fragments of *HSR201* with *mVenus* are underlined. The fluorescence pattern of HSR201 deletions is summarized on the right. PTS1, a fluorescent pattern similar to that of mVenus-PTS1. Intermediate, a fluorescent pattern that is intermediate between those of mVenus-PTS1 and mVenus only. (B) *Agrobacterium* cells carrying any one of the indicated mVenus-HSR201 deletions expressed from a modified 35S promoter were infiltrated into *N. benthamiana* leaves. Two days after infiltration, fluorescence was observed. Bar = 50 μm. (C) A schematic representation of the HSR201 mutants fused to mVenus. The fluorescence pattern of HSR201 mutants is summarized on the right. PTS1, a fluorescent pattern similar to that of mVenus-PTS1. YFP, a fluorescent pattern similar to that of mVenus only. Intermediate, a fluorescent pattern that is intermediate between those of mVenus-PTS1 and mVenus only. (D) *Agrobacterium* cells carrying any one of the indicated mVenus-HSR201 mutants expressed from a modified 35S promoter were infiltrated into *N. benthamiana* leaves. Two days after infiltration, fluorescence was observed. Bar = 50 μm.

HSR201 has neither a recognizable PTS1 nor PTS2 (**Fig. 1A**) and protein-targeting prediction programs predicted no specific subcellular localization for HSR201. To identify a non-canonical PTS of HSR201, we initially prepared a series of deletion constructs of HSR201 fused to mVenus and investigated their subcellular localization (**Supplementary Fig. S1**). The C-terminal half of HSR201 was sufficient for the targeting of mVenus to peroxisomes, whereas the deletion of the five C-terminal amino acid residues of HSR201 abolished its peroxisomal localization, indicating the presence of the PTS at the C-terminus of HSR201. To narrow down the position of the PTS, shorter sequences of the HSR201 C terminus were fused to mVenus and their subcellular localization was investigated (**Fig. 2A, B**, **Supplementary Fig. S2A**). The fluorescence patterns of mVenus-HSR201(347–460), -HSR201(404–460), -HSR201(431–460), -HSR201(441–460) and -HSR201(452–460) were similar to those of mVenus-HSR201 and mVenus-PTS1, suggesting that the C-terminal nonapeptide of HSR201 was sufficient to direct mVenus to peroxisomes (**Fig. 2B**, **Supplementary Fig. S2A**). Furthermore, the fluorescence pattern of mVenus-HSR201(458–460) was similar to that of mVenus alone; however, a few punctate fluorescence signals that were not present in the fluorescence pattern of mVenus alone were weakly observed (**Fig. 2B**). These results suggest that the C-terminal tripeptide of HSR201 functioned weakly than the full length and nonapeptide of HSR201.

### Identification of key amino acid residues for the peroxisomal targeting of HSR201

We compared the nine C-terminal amino acid residues of HSR201 with those of HSR201 homologs from various plant species (**Supplementary Fig. S2B**). Amino acid residues at positions −6, −5 and −3 to −1 from the C-terminal end were conserved compared with those at the other positions. The majority of amino acid residues were those with a hydrophobic side chain, such as Phe, Ile and Leu at positions −6, −5 and −1, Ser at position −3, and those with a small side chain, including Ala and Ser at position −2. The amino acid residues of HSR201 at these positions were identical or similar to the major ones, except for position −3 at which HSR201 had Pro instead of Ser. To identify the amino acid residues of HSR201 that are important for its peroxisomal targeting, each amino acid residue at positions −6 to −1 of HSR201 was initially replaced by Gly, a rare low abundance residue in PTS1 (Reumann et al. 2016, Deng et al. 2022), and their subcellular localization was observed (**Fig. 2C, D**, **Supplementary Fig. S2C**). The substitution of extreme C-terminal Leu^460^ completely compromised the peroxisomal targeting of mVenus-HSR201; however, the other mutations exerted no apparent effect. We then prepared HSR201 mutants in which two of the three amino acid residues at positions −6, −5 and −2 were converted to Gly because these residues were conserved between HSR201 and its homologs (**Supplementary Fig. S2B**). As shown in **Fig. 2D**, the substitution of Ala^459^ together with Ile^455^ or Ile^456^ partially compromised the peroxisomal targeting of mVenus-HSR201, whereas the substitution of both Ile^455^ and Ile^456^ exerted no apparent effect.

### PEX5, but not PEX7, is required for the peroxisomal localization of HSR201

**Fig. 3:**
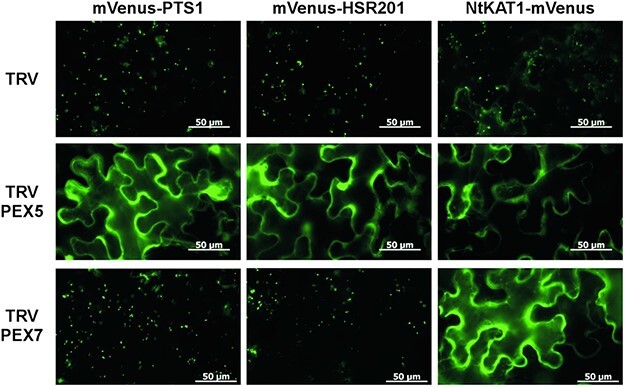
PEX5 is required for the peroxisomal localization of HSR201. *Nicotiana benthamiana* plants were infected with TRV (control), TRV:PEX5 or TRV:PEX7. Two weeks later, *Agrobacterium* cells carrying any one of the indicated mVenus-fused genes expressed from a modified 35S promoter were infiltrated into the leaves of TRV-infected *N. benthamiana* plants. Two days after infiltration, fluorescence was observed. Bar = 50 μm.

PTS1 and PTS2 are recognized by their cytosolic receptors PEX5 and PEX7, respectively (Kao et al. 2018). In plants, PEX5 functions as a co-receptor for PTS2 and is required for the peroxisomal targeting of PTS1- and PTS2-containing proteins. To investigate whether PEX5 and PEX7 are required for the peroxisomal localization of HSR201, we suppressed their expression by virus-induced gene silencing (VIGS) in *N. benthamiana* (**Supplementary Fig. S3A**). As previously reported in *Arabidopsis* (Hayashi et al. 2005), *PEX5*-suppressed plants had yellow-green leaves, whereas *PEX7*-suppressed plants did not have any apparent morphological phenotypes (**Supplementary Fig. S3B**). mVenus-HSR201 was expressed in the leaves of *PEX5*-silenced and *PEX7*-silenced plants as well as those of control plants. mVenus-PTS1 and NtKAT1-mVenus were used as markers of PTS1- and PTS2-containing proteins, respectively. As expected, mVenus-HSR201, mVenus-PTS1 and NtKAT1-mVenus mainly localized in the peroxisomes of control plants (**Fig. 3**). In contrast to mVenus-PTS1 and mVenus-HSR201, fluorescence from NtKAT1-mVenus was not exclusively restricted to peroxisomes and was also weakly observed in entire cells. The suppression of *PEX5* compromised the peroxisomal localization of mVenus-HSR201, mVenus-PTS1 and NtKAT1-mVenus, whereas the suppression of *PEX7* specifically compromised the peroxisomal localization of NtKAT1-mVenus. These results suggest that the peroxisomal localization of HSR201 is PEX5-dependent.

### The peroxisomal localization of HSR201 is required to enhance NtCNL-induced SA accumulation

**Fig. 4:**
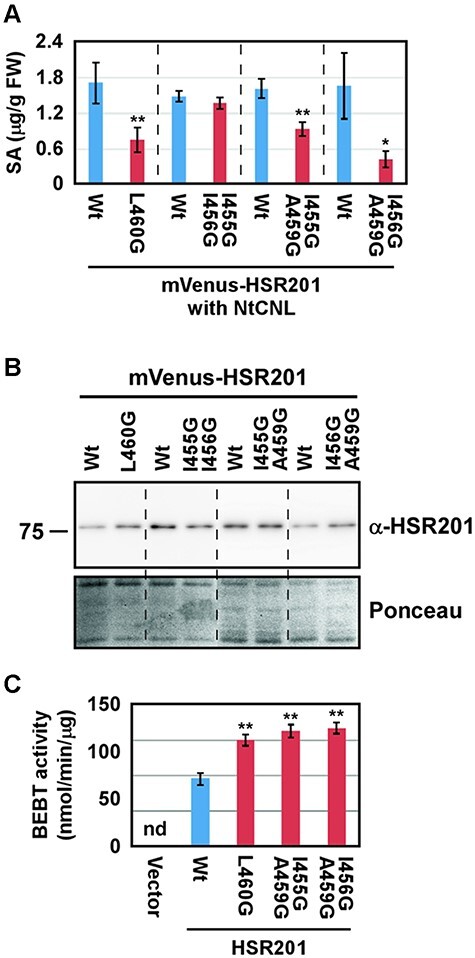
The peroxisomal localization of HSR201 is required to enhance NtCNL-induced SA accumulation. (A) *Agrobacterium* cells carrying *NtCNL* expressed from a modified 35S promoter were mixed with those containing any one of mVenus-HSR201 wild type, L460G, I455G/I456G, I455G/A459G and I456G/A459G expressed from the same promoter at a ratio of 1: 1, and the mix was then infiltrated into *N. benthamiana* leaves. Two days later, SA levels were measured. Values are the means with standard errors of four to eight biological replicates. The significance of differences between the HSR201 wild type and mutants was assessed using the Student’s *t*-test with Excel 2021 software (**P < 0.01, *P < 0.05). (B) *Agrobacterium* cells carrying any one of the indicated mVenus-HSR201 wild type and mutants were infiltrated into *N. benthamiana* leaves. Two days later, the accumulation of proteins was detected by immunoblotting analyses using an anti-HSR201 antibody (α-HSR201). As a loading control, blots were stained with Ponceau-S (Ponceau). Experiments were repeated four times with similar results. The position of the molecular mass marker in kilodaltons is indicated on the left. (C) Recombinant proteins of HSR201 wild type, L460G, I455G/A459G and I456G/A459G without any tag were expressed in *E. coli*. Soluble protein fractions were prepared from cells, and their BEBT activity was measured. Values are the means with standard deviations of three reactions. Significant differences from the wild type were assessed using a one-way ANOVA followed by Dunnett’s test with KaleidaGraph 5.0 software (**P < 0.01). The experiment was repeated twice with similar results using independently prepared soluble protein fractions. *E. coli* carrying an empty vector was used as a control (vector). nd, not detected.

The overexpression of *NtCNL* in *N. benthamiana* leaves was previously shown to induce the accumulation of SA, which was enhanced by the co-expression of HSR201 (Kotera et al. 2023). To investigate whether the peroxisome targeting of HSR201 was required to enhance NtCNL-induced SA accumulation, mVenus-HSR201 wild type and mutants were expressed together with NtCNL under the control of a strong constitutively active promoter using *Agrobacterium*-mediated transient expression. The effects of the HSR201 mutants, which showed non- or partial peroxisome targeting to enhance NtCNL-induced SA accumulation, were weaker than the wild type and similar to the vector control (**Fig. 4A**, **Supplementary Fig. S4A**). On the other hand, the effects of the HSR201 mutants, which targeted peroxisomes, were similar to the wild type (**Fig. 4A**, **Supplementary Fig. S4A**). These results indicate that the peroxisomal localization of HSR201 was required to enhance NtCNL-induced SA biosynthesis. However, amino acid substitutions sometimes decrease the stability of proteins, and the reduced enhancing activity of the HSR201 mutants may be attributed by their decreased protein levels. Therefore, the protein levels of the mVenus-HSR201 mutants that accumulated in *N. benthamiana* leaves were compared with those of the wild type. Although the protein levels of the mutants were sometimes slightly higher or slightly lower than those of the wild type during the repetition of the experiments, overall the stability of the mutants was similar to that of the wild type (**Fig. 4B**, **Supplementary Fig. S4B**). Amino acid substitutions may also have affected the enzymatic activity of HSR201, particularly if the substituted amino acid residue was involved in the catalysis or substrate binding. Therefore, the BEBT activities of the HSR201 mutants with non- or partial peroxisome targeting were compared with that of the wild type. Recombinant proteins of the HSR201 wild type and mutants were expressed in *Escherichia coli* without any tag because D’Auria et al. (2002) previously reported that the introduction of a His tag at the N- or C-terminal ends of the *Clarkia breweri* BEBT markedly decreased its activity. All the mutant HSR201 proteins were more soluble than the wild-type protein in *E. coli* cells (**Supplementary Fig. S4C**). The replacement of hydrophobic amino acid residues, such as leucin and isoleucine, with glycine may have contributed to the increased solubility of the mutant proteins. BEBT activity was measured using soluble protein fractions prepared from transformed cells because the soluble protein fraction from cells transformed with an empty vector did not exhibit any detectable activity (**Fig. 4C**). As shown in **Fig. 4C**, all the mutants exhibited higher BEBT activity than the wild type. These results suggest that peroxisomal localization was required for HSR201 to enhance NtCNL-induced SA accumulation.

### The peroxisomal localization of HSR201 is required for pathogen signal-induced SA accumulation

**Fig. 5:**
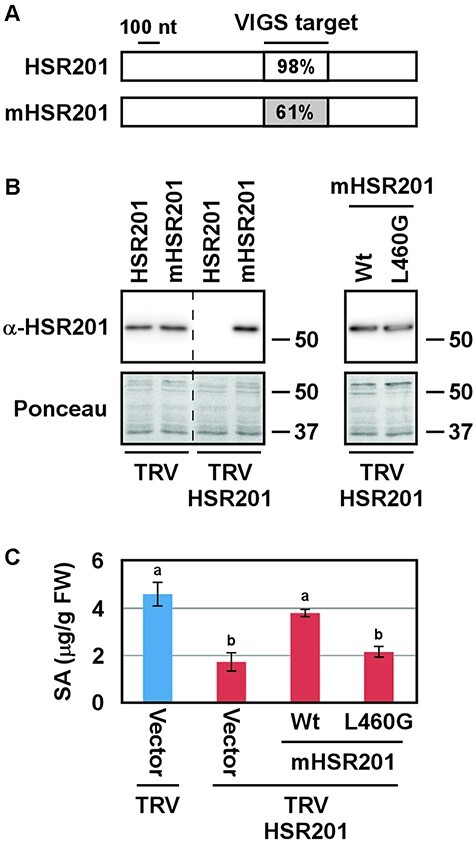
The peroxisomal localization of HSR201 is required for pathogen signal-induced SA accumulation. (A) A schematic representation of *HSR201* and *mHSR201*. (B) *Nicotiana benthamiana* plants were infected with TRV (control) or TRV:HSR201. Two weeks later, *Agrobacterium* cells carrying any one of the indicated *HSR201* genes expressed from a modified 35S promoter were infiltrated into the leaves of TRV-infected plants. Two days later, the accumulation of proteins was detected by immunoblotting analyses using an anti-HSR201 antibody (α-HSR201). As a loading control, blots were stained with Ponceau-S (Ponceau). The position of the molecular mass marker in kilodaltons is indicated on the right. Wt, wild type. Experiments were repeated twice with similar results. (C) *Nicotiana benthamiana* plants were infected with TRV (control) or TRV:HSR201. Two weeks later, *Agrobacterium* cells carrying *mHSR201* (Wt) or *mHSR201 L460G* expressed from a modified 35S promoter were infiltrated into the leaves of TRV-infected plants. *Agrobacterium* carrying an empty vector was used as a control (Vector). Two days later, INF1 solution (170 nM) was infiltrated into the same positions of the leaves. SA levels were measured 24 h after the INF1 treatment. Values are the means with standard errors of seven to twelve biological replicates. The significance of differences among the groups was assessed with a one-way ANOVA followed by Tukey’s HSD using KaleidaGraph 5.0 software. Lowercase letters above the bars indicate significant differences (*P* < 0.01).

We then investigated whether the peroxisomal localization of HSR201 was required for stress-induced SA biosynthesis. VIGS of *NbHSR201*, an *N. benthamiana* homolog of *HSR201*, has been shown to compromise SA production induced by INF1, an elicitor secreted by *Phytophthora infestans* (Kamoun et al. 1997, Takagi et al. 2022). Therefore, we examined whether the introduction of HSR201 L460G, which showed non-peroxisome targeting (**Fig. 2**), into *NbHSR201*-suppressed plants restored INF1-induced SA production. However, the nucleotide sequence of the *NbHSR201* fragment inserted into the viral vector was 98% identical to that of the corresponding region of *HSR201* (**Fig. 5A**, **Supplementary Fig. S5**), and *HSR201* introduced into *NbHSR201*-suppressed plants was silenced by VIGS (**Fig. 5B**). To overcome this issue, we introduced synonymous mutations into the VIGS target region of *HSR201*, which decreased its nucleotide sequence identity to the *NbHSR201* insert from 98% to 61% (**Fig. 5A**, **Supplementary Fig. S5**). This *HSR201* mutant, designated as *mHSR201*, escaped from VIGS and its protein accumulated in *NbHSR201*-suppressed plants (**Fig. 5B**). As was the case for the original HSR201, the L460G mutation did not affect the protein stability of mHSR201 (**Fig. 5B**). As shown in **Fig. 5C**, VIGS of *NbHSR201* compromised INF1-induced SA accumulation and the expression of mHSR201 wild type, but not mHSR201 L460G, complemented it. These results indicate that the peroxisomal localization of HSR201 was required for pathogen stress-induced SA accumulation.

### The non-canonical PTS1 of HSR201 functions in budding yeast but not in human cells

**Fig. 6:**
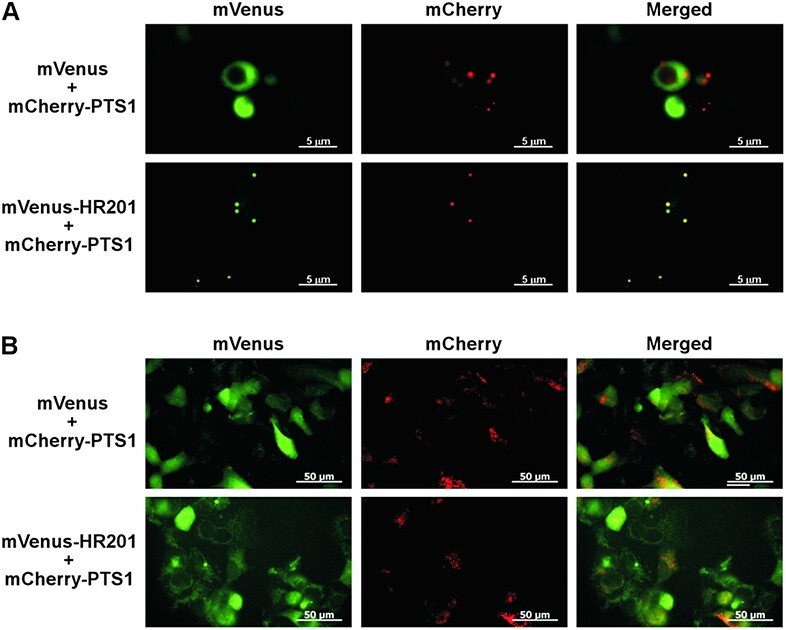
HSR201 is targeted to peroxisomes in budding yeast but not in human cells. (A) Yeast strain BY4741 carrying both mVenus-HSR201 expression plasmid and mCherry-PTS1 expression plasmid were cultured in a synthetic-defined medium supplemented with raffinose at 30°C and then the expression of mVenus-HSR201 was induced by galactose. Six hours after induction, the cells were washed and then fluorescence was observed. As a control, mVenus alone was expressed similarly. Bar = 5 μm. (B) HEK293 cells were transiently transfected with mVenus-HSR201 and mCherry-PTS1 expressed from an immediate early promoter of cytomegalovirus. As a control, mVenus alone was expressed in a similar manner. Sixteen hours after transfection, cells were fixed, washed and fluorescence was observed. Bar = 50 μm.

The import mechanisms of peroxisomal matrix proteins are evolutionarily conserved to a large extent in eukaryotes (Baker et al. 2016), and proteins with canonical PTS1, such as a firefly luciferase, are imported into the peroxisomes of yeasts, plants and mammals (Gould et al. 1990). To investigate whether the non-canonical PTS1 of HSR201 targeted mVenus to the peroxisomes of yeasts and mammals, mVenus-HSR201 was co-expressed with mCherry-PTS1 in budding yeast (*Saccharomyces cerevisiae*) and human (*Homo sapiens*) HEK293 cells. As a control, only mVenus was co-expressed with mCherry-PTS1. In budding yeast, *mVenus-HSR201* and *mVenus* were expressed from the galactose-inducible *GAL1* promoter (Lohr et al. 1995), whereas *mCherry-PTS1* was driven by the constitutive *PGK1* promoter (Packham et al. 1996). As shown in **Fig. 6A**, mVenus-HSR201 and mCherry-PTS1 showed similar punctate fluorescence patterns. In cells expressing both mVenus-HSR201 and mCherry-PTS1, their fluorescence patterns overlapped with each other; however, most of the cells only expressed one of them. On the other hand, fluorescence from mVenus alone was observed throughout cells and did not overlap with fluorescence from mCherry-PTS1. In human HEK293 cells, *mVenus-HSR201, mVenus* and *mCherry-PTS1* were expressed under the control of an immediate early promoter of cytomegalovirus (Stinski and Isomura 2008). As shown in **Fig. 6B**, neither mVenus nor mVenus-HSR201 showed specific subcellular localization and their fluorescence patterns did not overlap with that of mCherry-PTS1, which showed a typical punctate fluorescence pattern.

## Discussion

We herein demonstrated that BEBT HSR201 mainly localized in peroxisomes via its non-canonical PTS in *N. benthamiana* leaves, which was required for SA biosynthesis induced by a pathogen-derived signal as well as by the overexpression of *NtCNL* (**Figs. 1**, **2**, **4**, **5**, **Supplementary Fig. S4**). We previously reported that the peroxisomal β-oxidative pathway proteins NtCNL, NtCHD and NtKAT1 also mainly localized in peroxisomes, converted CA to BA-CoA in vitro and contributed to SA biosynthesis cooperatively with HSR201 (Kotera et al. 2023). These findings and the present results suggest that HSR201 converts BA-CoA or its derivatives produced by the peroxisomal β-oxidative pathway to benzyl benzoate or its derivatives in peroxisomes. Benzyl benzoate and its derivatives are regarded as important intermediates in the biosynthesis of benzenoid compounds in plant species, such as petunia (*Petunia hybrida*) and poplar (*Populus trichocarpa*) (Orlova et al. 2006, Fellenberg et al. 2020, Gordon et al. 2022). In these plant species, benzyl benzoate and its derivatives were proposed to be produced in the cytosol because their HSR201 homologs (PhBPBT in petunia and PtSABT and PtBEBT in poplar) had no apparent target peptides or presequences, including PTSs (Boatright et al. 2004, Chedgy et al. 2015, Adebesin et al. 2018). We compared the C-terminal amino acid sequence of HSR201 with those of the homologs (**Supplementary Fig. S6**). Our mutational analysis identified one essential (Leu^460^ at position −1) and three important (Ile^455^, Ile^456^ and A^459^ at positions −6, −5 and −2, respectively) amino acid residues of HSR201 for localization in peroxisomes (**Fig. 2**). Amino acid sequence alignment showed that these residues were highly conserved in homologs with a consensus pattern of hydrophobic amino acid residues at positions −6 and −5 and amino acid residues with a small side chain at position −2 and Leu at position −1. This result strongly suggests that the petunia and poplar homologs are also targeted to peroxisomes and, thus, benzyl benzoate and its derivatives are produced in peroxisomes. In addition to HSR201 and the peroxisomal β-oxidative pathway proteins, HSR203J, another hypersensitivity-related protein, is required for pathogen signal-induced SA biosynthesis (Takagi et al. 2022). The physiological substrate of HSR203J is currently unknown; however, its recombinant protein exhibits esterase activity toward artificial substrates (Baudouin et al. 1997). Further studies are needed to clarify whether HSR203J hydrolyzes benzyl benzoate into benzoic acid and benzyl alcohol. HSR203J possesses no apparent target peptides or presequences and its subcellular localization has not been reported. The subcellular localization of HSR203J will be an important subject of future analyses.

The PTS of HSR201 was positioned at its C-terminal end (**Fig. 2**) and VIGS of *PEX5*, a PTS1 receptor, but not *PEX7*, a PTS2 receptor, compromised the peroxisomal targeting of HSR201 (**Fig. 3**). These results indicate that the PTS of HSR201 is a variant of PTS1. Canonical PTS1 is a tripeptide located at the C-terminal end of proteins with the consensus sequence of [S/A]-[K/R]-[L/M/I]. PEX5 binds to PTS1 via its C-terminal domain containing seven tetratricopeptide repeats (Reumann et al. 2016). The X-ray structures of the C-terminal domain of PEX5 proteins from human and *Trypanosoma brucei* complexed with model peptides containing canonical PTS1 sequences revealed the molecular mechanisms underlying the interaction between PEX5 and PTS1 peptides (Gatto et al. 2000, Sampathkumar et al. 2008). The hydrophobic side chain of residues at position −1 form hydrophobic interactions with the tetratricopeptide repeats of PEX5. The positively charged side chain of residues at position −2 is located within a negatively charged pocket where it hydrogen bonds to negatively charged residues of PEX5 directly or via a water molecule. The small side chain of residues at position −3 hydrogen bonds to the receptor indirectly via water. Except for Leu^460^ at position −1, the properties of the amino acid residues of HSR201 at positions −3 to −1 differed from those of the canonical PTS1s (**Fig. 1**). Consistently, the introduction of a single mutation into Leu^460^, but not either of the other two residues (Pro^458^ and Ala^459^), compromised the peroxisomal localization of HSR201 (**Fig. 2C, D**, **Supplementary Fig. S2C**). The sequences upstream of the PTS1 tripeptide have been shown to affect the peroxisome targeting ability of PTS1 (Reumann et al. 2016). Deng et al. (2022) systematically analyzed the effects of the upstream sequences on PTS1-mediated peroxisomal targeting and revealed that the amino acid residues with a ‘basic-nonpolar-basic’ pattern at positions −6 to −4 exerted strong enhancing effects. Although the amino acid residue of HSR201 at position −6 is not basic (Ile^455^), those at positions −5 and −4 are nonpolar (Ile^456^) and basic (Arg^457^). However, the introduction of a single mutation into Ile^456^ or Arg^457^ did not significantly affect the peroxisomal localization of HSR201 (**Fig. 2C, D**, **Supplementary Fig. S2C**). The mutation of Ile^456^ weakened the peroxisomal targeting of HSR201 only when Ala^459^ was also mutated. HSR201 was imported into the peroxisomes of *N. benthamiana* leaves and budding yeast, but not human cells (**Figs. 1**, **6**). On the other hand, proteins with canonical PTS1 have been imported into the peroxisomes of yeasts, plants and mammals (Gould et al. 1990). Consistently, the C-terminal halves of budding yeast and methylotrophic yeast (*Pichia pastoris*) PEX5 were shown to be interchangeable with those of plant and human PEX5, respectively, in the peroxisomal import of PTS1-containing proteins (Wiemer et al. 1995, Kragler et al. 1998). Collectively, these findings indicate that PEX5 recognizes the PTS of HSR201 in a different manner from canonical PTS1. These results also suggest that *N. benthamiana* and yeast PEX5, but not human PEX5 can recognize the PTS of HSR201 although we cannot rule out the possibility that HSR201 was not folded correctly in human cells and thus not imported into peroxisomes. In the future, we will compare the molecular mechanisms underlying the interactions with PEX5 between the PTS of HSR201 and canonical PTS1.

Non-canonical PTS1-mediated peroxisomal localization was important for HSR201 to contribute to SA biosynthesis (**Figs. 4**, **5**, **Supplementary Fig. S4**); however, the physiological importance of having a non-canonical PTS1, but not a canonical PTS1, remains unknown. Although a previous study demonstrated that many proteins were imported into peroxisomes via non-canonical PTS1s (Reumann et al. 2016), their significance remains unclear in most cases. A rare example for which the importance of having a non-canonical PTS1 has been clarified is human catalase, a hydrogen peroxide-decomposing enzyme. Human catalase harbors an atypical PTS1, KANL, at its C terminus and is imported into peroxisomes by a PEX5-mediated pathway, similar to typical PTS1 proteins, under non-stressed conditions (Purdue and Lazarow 1996). Oxidative stress, such as a hydrogen peroxide treatment, has been shown to induce the phosphorylation of PEX14, a central component of the protein translocation complex in peroxisomal membranes (Okumoto et al. 2020). The phosphorylation of PEX14 selectively suppresses the peroxisomal import of catalase, but not canonical PTS1 proteins, which increases the levels of cytosolic catalase to counteract oxidative stress while maintaining the homeostasis of peroxisomes. Since the rapid production of SA is important to cope with stress conditions, particularly pathogen infections, HSR201 may be targeted to peroxisomes more preferentially than canonical PTS1 proteins under stress conditions.

In the present study, we showed that BEBT HSR201 mainly localized in peroxisomes via its non-canonical PTS1 in *N. benthamiana* leaves and budding yeast. The non-canonical PTS1-mediated peroxisomal localization of HSR201 was required for SA biosynthesis in *N. benthamiana* leaves; however, the importance of carrying a non-canonical PTS1, but not a canonical PTS1, is unknown. By elucidating the importance and mechanisms of the non-canonical PTS1-mediated peroxisomal localization of HSR201, a more detailed understanding of the regulatory mechanisms underlying the biosynthesis of not only SA, but also benzenoid compounds produced from benzyl benzoate will be obtained.

## Materials and Methods

### Plant growth conditions

*Nicotiana benthamiana* were grown in a chamber maintained at 25˚C with 24 h of light until the four-true-leaf stage, and then the plants were grown in the same chamber with a 16 h light/25˚C and 8 h dark/20˚C cycle.

### Bioinformatics analysis

Subcellular localization of HSR201 was predicted by using Plant-mPLoc (http://www.csbio.sjtu.edu.cn/bioinf/plant-multi/) as described previously (Kotera et al. 2023). To create a Web-logo image (Crooks et al. 2004), the nine C-terminal amino acid sequences of HSR201 homologs (**Supplementary Table S1**) were subjected to WebLogo 3 (https://weblogo.threeplusone.com).

### Genes and plasmid vectors

The coding sequences of *HSR201, NtCNL* and *NtKAT1* were previously cloned in our group (Kotera et al. 2023). Synthetic *mVenus* and *mCherry* genes of which codons are optimized for the expression in tobacco were produced previously (Kotera et al. 2023). The binary vector pEl2Ω (Ohtsubo et al. 1999) was used for expression in *N. benthamiana* leaves. The pRS313 (U03439) vector containing *PGK1* promoter and the pRS315 (U03441) vector containing *GAL1* promoter were used for expression in budding yeast (Spear and Ng 2003). The pEGFP-N1 vector (U55762.1) was used for expression in human HEK293 cells.

### Construction of the vectors for the expression in *N. benthamiana* leaves, yeast and human HEK293 cells

For the expression of mVenus-HSR201 in *N. benthamiana* leaves, the *mVenus* was inserted into *Xba*I and *Bam*HI sites of the pEl2Ω vector and then the coding sequence of *HSR201* was cloned into *Bam*HI and *Spe*I sites of the same vector. For the expression of mVenus-HSR201 with deletions, gene fragments of *HSR201* were amplified by PCR or created by annealing oligonucleotide pairs and then fused to *mVenus* in the same manner as mVenus-HSR201. For the expression of mVenus-HSR201 with mutations, the coding sequences of *HSR201* were amplified by PCR using primers with mutations and then fused to *mVenus* in the same manner as mVenus-HSR201. The construction of pEl2Ω-NtCNL, -NtKAT1-mVenus, -mVenus-PTS1 and -mCherry-PTS1 vectors has been described previously (Kotera et al. 2023). For expression in budding yeast, *mVenus-HSR201* and the pRS315 vector bearing *GAL1* promoter were amplified by PCR and fused using the In-Fusion HD cloning kit (Takara Bio Inc., Shiga, Japan). In similar ways, *mVenus* and *mCherry-PTS1* were fused to the pRS315 vector bearing *GAL1* promoter and the pRS313 vector bearing *PGK1* promoter, respectively. For expression in human HEK293 cells, *mVenus, mVenus-HSR201* and *mCherry-PTS1* were amplified by PCR and cloned into *Sma*I and *Not*I sites of the pEGFP-N1 vector in place of *green fluorescent protein* (*GFP*) using the In-Fusion HD cloning kit (Takara Bio Inc., Shiga, Japan). Primer and oligonucleotide pairs are listed in **Supplementary Table S2**.

### *Agrobacterium*-mediated transient expression

Transformation, culture and preparation of *Agrobacterium* (strain GV3101) cells were performed as described previously (Yokoo et al. 2018). In the analyses of subcellular localization, protein levels and complementation, *Agrobacterium* cells (OD_600_ = 0.1) were infiltrated into *N. benthamiana* leaves. In the analyses of VIGS and SA induction, *Agrobacterium* cells (OD_600_ = 0.5) were infiltrated into leaves.

### Subcellular localization analysis in *N. benthamiana* leaves

Subcellular localization analysis in *N. benthamiana* leaves was performed as described previously (Kotera et al. 2023).

### Subcellular localization analysis in yeast cells

Yeast strain BY4741 bearing both mVenus expression plasmid and mCherry-PTS1 expression plasmid, or mVenus-HSR201 expression plasmid and mCherry-PTS1 expression plasmid was cultured in 1 ml of synthetic defined medium supplemented with raffinose at 30°C for around 16 h. After the cultivation, 2 ml of synthetic defined medium supplemented with galactose was added and the cells were further incubated at 30°C for 6 h. After the incubation, the cells were collected using a brief centrifugation, resuspended in distilled water and then fluorescence from mVenus and mCherry was observed as described previously (Kotera et al. 2023).

### Subcellular localization analysis in human HEK293 cells

HEK293 cells were cultured in Dulbecco’s Modified Eagle’s Medium (D7777; Merck KGaA, Darmstadt, Germany) supplemented with 10% fetal bovine serum (SH30396.03; Cytiva, Tokyo, Japan) and 1% penicillin–streptomycin mixed solution (09367–34; Nacalai Tesque Inc., Kyoto, Japan). Cells were incubated at 37°C under 5% CO_2_ and passaged before grown to 95% confluence. For transfection, the cells were seeded on 8-well µ-slide Removable Chamber (80,841; ibidi GmbH, Gräfelfing, Germany) and transfected with plasmids when the cells were grown to 80% confluence. Transfection was performed using Lipofectamine® 3000 Transfection Kit (L3000-015; Thermo Fisher Scientific, Waltham, MA, USA) in accordance with the manufacturer’s recommendations. Briefly, 60 ng of each plasmid were mixed with Opti-MEM®I (31,985–070; Thermo Fisher Scientific, Waltham, MA, USA) and lipofectamine 3000 reagent, and added per well. After incubation at 37°C under 5% CO_2_ for 16 h, the cells were fixed with 4% paraformaldehyde, washed with 1 × phosphate buffered saline and then fluorescence from mVenus and mCherry was observed as described previously (Kotera et al. 2023).

### Virus-induced gene silencing

The *N. benthamiana* genes corresponding to *Arabidopsis PEX5* (At5g56290) and *PEX7* (At1g29260) were identified by BLAST search using the Sol Genomics Network database (http://solgenomics.net/). The Sol Genomics Network ID numbers are Niben101Scf03733g00011.1 and Niben101Scf17372g00014.1 for *PEX5*, and Niben101Scf05484g01003.1 and Niben101Scf00279Ctg074 for *PEX7*. The pTV00 vectors containing the gene fragments of *PEX5* and *PEX7* were constructed as described previously (Takagi et al. 2022). The pTV00 vector for *NbHSR201* has been reported previously (Takagi et al. 2022). VIGS was performed as described previously (Ratcliff et al. 2001, Takagi et al. 2022). Briefly, the second to fourth true leaves of plants at the four-true-leaf stage were inoculated with virus. Two weeks later, the sixth and seventh leaves of the inoculated plants were used for experiments. Primer pairs used to amplify the fragments of *PEX5* and *PEX7* are listed in **Supplementary Table S2**.

### RNA extraction and RT-qPCR analysis

The extraction of total RNA and RT-qPCR analysis were performed as described previously (Katou et al. 2013). Primer pairs are listed in **Supplementary Table S2**.

### SA measurement

The extraction and quantification of SA were performed as described previously (Malamy et al. 1992, Takagi et al. 2022).

### Production and purification of an anti-HSR201 antibody

Production of anti-HSR201 antisera was performed as described previously (Kojima et al. 2019). Briefly, the peptide (AKPTPRETKFLSDIDDQEG) corresponding to residues 24–42 of HSR201 was synthesized, conjugated to keyhole limpet hemacyanin carrier and used to raise polyclonal antisera in rabbits by Cosmo Bio Co., Ltd. (Tokyo, Japan). Purification of antibodies from antisera was performed as follows. The coding sequence of *HSR201* was cloned into *Bam*HI and *Not*I sites of a pET28a vector (Merck KGaA, Darmstadt, Germany), allowing the production of N-terminal His_6_-tagged HSR201 (His-HSR201). Transformation of *E. coli* strain Rosetta2(DE3) (Merck KGaA, Darmstadt, Germany) and the induction of recombinant protein were performed as described previously (Takagi et al. 2022). The production of recombinant proteins was induced with 0.1 mM IPTG at 20˚C overnight and purified with a 1 ml HisTrap HP column (Cytiva, Tokyo, Japan) in accordance with the manufacturer’s recommendations. Purified His-HSR201 protein (∼0.4 mg) was separated on a SDS-polyacrylamide gel and transferred to polyvinylidene difluoride membranes (Merck KGaA, Darmstadt, Germany). After blocking with 5% non-fat dry milk, the membranes were washed with TBS-T (20 mM Tris-HCl, pH 7.5, 150 mM NaCl and 0.05% Tween 20) and 150 mM NaCl. And then the membranes were incubated in 4 ml of antisera at room temperature overnight. After washing with TBS-T and 150 mM NaCl, bound antibodies were eluted with 0.1 M Glycine-HCl, pH 2.5, immediately neutralized and concentrated using an Amicon Ultra-4 (Merck KGaA, Darmstadt, Germany).

### Protein extraction and immunoblotting analysis

Protein extraction from *N. benthamiana* leaves and immunoblotting analysis were performed as described previously (Kojima et al. 2019). As primary antibodies, 0.06 μg/ml rabbit polyclonal anti-HSR201 antibody and 0.1 μg/ml mouse monoclonal anti-GFP antibody (clone 1E4, Medical & Biological Laboratories, Tokyo, Japan) in 1% nonfat dry milk were used.

### Expression of recombinant HSR201 proteins in *E. coli* and BEBT activity assay

The coding sequences of *HSR201* and its mutants were cloned into *Nco*I and *Not*I sites of the pET28a vector (Merck KGaA, Darmstadt, Germany) to produce their recombinant proteins without any tag. Transformation of *E. coli* strain Rosetta2(DE3) (Merck KGaA, Darmstadt, Germany) and the induction of recombinant proteins were performed as described previously (Takagi et al. 2022). The production of recombinant proteins was induced with 0.1 mM IPTG at 20˚C overnight. After a culture, the cells were collected using a brief centrifugation, resuspended in 50 mM Tris-HCl, pH 7.5 and the crude protein fractions were prepared by sonication. After centrifugation of the crude protein fractions, the supernatants were used as a soluble protein fraction. The crude and soluble protein fractions were separated on a SDS-polyacrylamide gel and stained with Coomassie brilliant blue. Concentration of recombinant proteins was estimated by comparison with known concentration of molecular mass markers (Bio-Rad Laboratories, Inc. Hercules, CA, USA) on the gel. After dilution with 50 mM Tris-HCl, pH 7.5, the soluble protein fractions were incubated in 50 mM Tris-HCl, pH 7.5, 0.5 mM BA-CoA and 1 mM BA-alcohol at 30˚C for 5–10 min. To measure the activity in a linear range, concentration of recombinant proteins in the reaction mixture was kept lower than 50 ng/ml. The reaction was terminated by diluting 10 times with 80% methanol. Reaction products were filtered through a 0.2 μm Millex-LG filter (Merck KGaA, Darmstadt, Germany) and then injected into an HPLC system with a photodiode array detector (Shimadzu Corp., Kyoto, Japan). The reaction products were separated on an octadecyl-silica column (μ-Bondasphere C18, 150 mm × ID 3.9 mm, 5 μm, 100A; Waters, Milford, MA, USA) maintained at 30°C with a flow rate of 1 ml/min using 70% methanol. The spectrum of the peaks was monitored from 190 nm to 600 nm. Benzyl benzoate was eluted around 8.5 min after injection and its amount was calculated based on the absorbance at 230 nm with commercial benzyl benzoate (FUJIFILM Wako Pure Chemical Corp., Osaka, Japan) as a standard.

### INF1 treatment

Preparation and treatment of INF1 were performed as described previously (Kamoun et al. 1997, Shibata et al. 2010).

### Statistical analysis

Student’s *t*-test was used with Excel 2021 software to determine a significant difference between two samples. One-way analysis of variance (ANOVA) followed by Dunnett’s test was used with KaleidaGraph 5.0 software to compare multiple samples with one fixed control. ANOVA followed by Tukey’s HSD was used with KaleidaGraph 5.0 software to determine significant differences among the groups. A *P*-value < 0.05 was considered statistically significant.

## Supplementary Material

pcae129_Supp

## Data Availability

The data underlying this article are available upon request.
