# Supplementary material for: Peroxisomal Localization of Benzyl Alcohol O-Benzoyltransferase HSR201 is Mediated by a Non-canonical Peroxisomal Targeting Signal and Required for Salicylic Acid Biosynthesis
Source: Plant Cell Physiol. 2024 Oct 29;65(12):2054–65. doi: 10.1093/pcp/pcae129 (PMC11662444; doi:10.1093/pcp/pcae129)
Supplement: pcae129_Supp [file pcae129_supp.zip › suppl_data/pcp-2024-e-00210-File008.pdf]

Supplementary Table S1 List of HSR201 homologs

Spiecies	Protein name	ID
Nicotiana tabacum	benzyl alcohol O-benzoyltransferase	NP_001312867
Petunia x hybrida	BEBT1	Q6E593
Solanum pennellii	benzyl alcohol O-benzoyltransferase	XP_015080810
Capsicum annuum	benzyl alcohol O-benzoyltransferase	XP_016580134
Ipomoea nil	benzyl alcohol O-benzoyltransferase-like	XP_019159544
Camellia sinensis	benzyl alcohol O-benzoyltransferase	XP_028089718
Actinida chinensis var. chinensis	Benzyl alcohol O-benzoyltransferase	PSS04601
Coffea eugenioides	benzyl alcohol O-benzoyltransferase-like	XP_027162010
Salvia splendens	benzyl alcohol O-benzoyltransferase-like	XP_042040152
Plectranthus barbatus	acyltransferase ACT8	AMZ03414
Lavandula x intermedia	alcohol acetyltransferase	AIW81431
Sesamum indicum	benzyl alcohol O-benzoyltransferase-like	XP_011085895
Dorcoceras hygrometricum	benzyl alcohol O-benzoyltransferase-like	KZV53802
Scoparia dulcis	benzoyl-CoA transferase 1	BCK60966
Olea europaea var. sylvestris	benzyl alcohol O-benzoyltransferase	XP_022858860
Phtheirospermum japonicum	benzyl alcohol o-benzoyltransferase	GFP82373
Striga asiatica	HXXXD-type acyl-transferase family protein	GER47056
Erythranthe guttata	benzyl alcohol O-benzoyltransferase-like	XP_012832498
Glandularia x hybrida	benzoyl CoA benzoic acid benzoyltransferase	BAE72881
Helianthus annuus	benzyl alcohol O-benzoyltransferase	XP_021985137
Lactuca sativa	benzyl alcohol O-benzoyltransferase	XP_023766374
Cynara cardunculus var. scolymus	benzyl alcohol O-benzoyltransferase-like	XP_024983320
Artemisia annua	benzyl alcohol O-benzoyltransferase	PWA57331
Tanacetum cinerariifolium	benzyl alcohol O-benzoyltransferase-like	GEV93276
Daucus carota subsp. sativus	benzyl alcohol O-benzoyltransferase-like	XP_017233968
Prunus dulcis	benzyl alcohol O-benzoyltransferase	XP_034227997
Fragaria vesca subsp. Vesca	benzyl alcohol O-benzoyltransferase	XP_004290447
Malus domestica	benzyl alcohol O-benzoyltransferase-like	XP_008359502
Rosa chinensis	benzyl alcohol O-benzoyltransferase	XP_024166499
Prunus yedoensis var. nudiflora	methanol O-anthraniloyltransferase	PQQ18992
Pyrus communis	alcohol acyl transferase	AAS48090
Pyrus ussuriensis	alcohol acyltransferase	AJD18611
Ziziphus jujuba	benzyl alcohol O-benzoyltransferase-like	XP_015899457
Trema orientale	Transferase	PON43845
Cannabis sativa	benzyl alcohol O-benzoyltransferase	XP_030484264
Morus notabilis	benzyl alcohol O-benzoyltransferase	XP_010108951
Benincasa hispida	benzyl alcohol O-benzoyltransferase	XP_038901429
Momordica charantia	benzyl alcohol O-benzoyltransferase-like	XP_022148522
Cucumis melo	benzyl alcohol O-benzoyltransferase-like	NP_001315395
Cucurbita maxima	benzyl alcohol O-benzoyltransferase-like	XP_023007354
Cucurbita moschata	benzyl alcohol O-benzoyltransferase-like	XP_022948214
Juglans regia	benzyl alcohol O-benzoyltransferase-like	XP_018828167
Juglans microcarpa x Juglans regia	benzyl alcohol O-benzoyltransferase	XP_041009724
Morella rubra	Benzyl alcohol O-benzoyltransferase	KAB1205273
Quercus lobata	benzyl alcohol O-benzoyltransferase-like	XP_030935542
Lupinus angustifolius	benzyl alcohol O-benzoyltransferase-like	XP_019413732
Lupinas albus	putative benzyl alcohol O-benzoyltransferase	KAE9622004
Cajanus cajan	benzyl alcohol O-benzoyltransferase	XP_020235949
Abrus precatorius	benzyl alcohol O-benzoyltransferase	XP_027365767
Prosopis alba	benzyl alcohol O-benzoyltransferase	XP_028800205
Glycine soja	benzyl alcohol O-benzoyltransferase-like	XP_028191632
Arachis hypogaea	benzyl alcohol O-benzoyltransferase	XP_025615216
Medicago truncatula	benzyl alcohol O-benzoyltransferase	XP_024641644
Clitoria ternatea	putative acyltransferase	BAF49304
Senna tora	benzyl alcohol O-benzoyltransferase	KAF7825022
Vigna unguiculata	benzyl alcohol O-benzoyltransferase	XP_027937129
Vigna angularis	benzyl alcohol O-benzoyltransferase	XP_017413409
Trifolium pratense	benzyl alcohol O-benzoyltransferase-like protein	PNX79555

Mucuna pruriens	Benzyl alcohol O-benzoyltransferase, partial	RDY09997
Spatholobus suberectus	Benzyl alcohol O-benzoyltransferase	TKY45075
Celastrus angulatus	BAHD acyltransferase	QLI57449
Manihot esculenta	benzyl alcohol O-benzoyltransferase-like	XP_021622269
Ricinus communis	benzyl alcohol O-benzoyltransferase-like	XP_002524021
Hevea brasiliensis	benzyl alcohol O-benzoyltransferase-like	XP_021662621
Jatropha curcas	benzyl alcohol O-benzoyltransferase	XP_012070283
Populus davidiana	benzoyl-CoA:benzyl alcohol O-benzoyltransferase	ARV79885
Populus trichocarpa	benzyl alcohol O-benzoyltransferase	XP_002325454
Populus euphratica	benzyl alcohol O-benzoyltransferase-like	XP_011012995
Salix suchowensis	benzyl alcohol O-benzoyltransferase	KAG5224345
Cephalotus follicularis	Transferase domain-containing protein	GAV70647
Corchorus olitorius	Transferase	OMP08049
Hibiscus syriacus	benzyl alcohol O-benzoyltransferase-like	XP_039028389
Herrania umbratica	benzyl alcohol O-benzoyltransferase-like	XP_021282796
Gossypium raimondii	benzyl alcohol O-benzoyltransferase-like	XP_012464383
Gossypium hirsutum	benzyl alcohol O-benzoyltransferase	XP_016726282
Corchorus capsularia	Transferase	OMO87278
Citrus sinensis	benzyl alcohol O-benzoyltransferase	XP_006484694
Pistacia vera	benzyl alcohol O-benzoyltransferase-like	XP_031262587
Syzygium oleosum	benzyl alcohol O-benzoyltransferase	XP_030472300
Punica granatum	benzyl alcohol O-benzoyltransferase	XP_031377852
Clarkia breweri	benzoyl coenzyme A: benzyl alcohol benzoyl transferase	AAN09796
Vitis vinifera	benzyl alcohol O-benzoyltransferase	XP_002264599
Vitis labrusca	anthraniloyal-CoA: methanol anthraniloyal transferase	AAW22989
Eutrema salsugineum	(Z)-3-hexen-1-ol acetyltransferase	XP_006400294
Vasconcellea cundinamarcensis	alcohol acyltransferase	ACT82248
Carica papaya	benzyl alcohol O-benzoyltransferase-like	XP_021911306
Dichanthelium oligosanthes	Benzyl alcohol O-benzoyltransferase	OEL15119
Setaria viridis	benzyl alcohol O-benzoyltransferase	XP_034570834
Zea mays	10-deacetylbaccatin III 10-O-acetyltransferase	NP_001152518
Sorghum bicolor	benzyl alcohol O-benzoyltransferase	XP_002466979
Panicum miliaceum	benzyl alcohol O-benzoyltransferase	RLN19241
Hordeum vulgare	benzyl alcohol O-benzoyltransferase	KAE8772172
Triticum dicoccoides	benzyl alcohol O-benzoyltransferase-like	XP_037408879
Brachypodium distachyon	benzyl alcohol O-benzoyltransferase	XP_003574156
Oryza sativa Japonica Group	benzyl alcohol O-benzoyltransferase	XP_015614459
Ananas comosus	benzyl alcohol O-benzoyltransferase-like	XP_020093248
Carex littledalei	benzyl alcohol O-benzoyltransferase	KAF3321584
Cocos nucifera	Benzyl alcohol O-benzoyltransferase	KAG1362783
Phoenix dactylifera	benzyl alcohol O-benzoyltransferase-like	XP_038982859
Elaeis guineensis	benzyl alcohol O-benzoyltransferase	XP_010941428
Musa acuminata subsp. malaccensis	benzyl alcohol O-benzoyltransferase	XP_009388282
Zingiber officinale	benzyl alcohol O-benzoyltransferase-like	XP_042377307
Apostasia shenzhenica	Benzyl alcohol O-benzoyltransferase	PKA45699
Dendorobium catenatum	Benzyl alcohol O-benzoyltransferase	PKU81747
Phalaenopsis equestris	benzyl alcohol O-benzoyltransferase	XP_020577420
Vanda hybrid cultivar	acyltransferase	ABX57719
Dioscorea cayenensis subsp. rotundata	benzyl alcohol O-benzoyltransferase-like	XP_039121200
Zostera marina	putative Benzyl alcohol O-benzoyltransferase	KMZ62258
Papaver somniferum	benzyl alcohol O-benzoyltransferase-like	XP_026401712
Macleaya cordata	Transferase	OVA18375
Nelumbo nucifera	benzyl alcohol O-benzoyltransferase	XP_010275172
Macadamia integrifolia	benzyl alcohol O-benzoyltransferase	XP_042504028
Beta vulgaris subsp. Vulgaris	benzyl alcohol O-benzoyltransferase	XP_010672751
Chenopodium quinoa	benzyl alcohol O-benzoyltransferase-like	XP_021738744
Spinacia oleracea	benzyl alcohol O-benzoyltransferase-like	XP_021852926
Cinnamomum micranthum f. kanehirae	benzyl alcohol O-benzoyltransferase	RWR87240
Nymphaea colorata	benzyl alcohol O-benzoyltransferase	XP_031482357
Amborella trichopoda	benzyl alcohol O-benzoyltransferase	XP_011623977
