# Supplementary material for: Peroxisomal Localization of Benzyl Alcohol O-Benzoyltransferase HSR201 is Mediated by a Non-canonical Peroxisomal Targeting Signal and Required for Salicylic Acid Biosynthesis
Source: Plant Cell Physiol. 2024 Oct 29;65(12):2054–65. doi: 10.1093/pcp/pcae129 (PMC11662444; doi:10.1093/pcp/pcae129)
Supplement: pcae129_Supp [file pcae129_supp.zip › suppl_data/pcp-2024-e-00210-File009.pdf]

Supplementary Table S2 List of primers used in this study

Construct	Forward	Reverse
Primer pairs for the construction of the pEI2Ω vectors[a,b]		
mVenus-HSR201	CGGGATCCATGGATTCAAAGCAATCATCAG	GACTAGTCAAAGGGCAGGTCTGATAATGGC
mVenus-HSR201-N	CGGGATCCATGGATTCAAAGCAATCATCAG	AACTAGTCAGTCGTCTAAGGGGATAATTGTACC
mVenus-HSR201-C	TAGGATCCATGGTTCACAAATCTTTCTTTTTTGGC	GACTAGTCAAAGGGCAGGTCTGATAATGGC
mVenus-HSR201 ΔN	TAGGATCCATGGGAAGGAAAGATCCTGTAAAGG	GACTAGTCAAAGGGCAGGTCTGATAATGGC
mVenus-HSR201 ΔC	CGGGATCCATGGATTCAAAGCAATCATCAG	AACTAGTCAAATGGCATAGTTTGAGTTGACTAATG
HSR201(347-460)	CGGGATCCATGAAATCTGTGGCAGATTTA	GACTAGTCAAAGGGCAGGTCTGATAATGGC
HSR201(404-460)	CGGGATCCGTGGCTAGTTTTTATATACCATTTAA	GACTAGTCAAAGGGCAGGTCTGATAATGGC
HSR201(431-460)	CGGGATCCATGGAAACATTCGTCAAAGAACT	GACTAGTCAAAGGGCAGGTCTGATAATGGC
HSR201(441-460)	CGGGATCCATGTTGAAAGTTGATGCTCCATTAGTC	GACTAGTCAAAGGGCAGGTCTGATAATGGC
HSR201 I455G	CGGGATCCATGGATTCAAAGCAATCATCAG	AACTAGTCAAAGGGCAGGTCTGATACCGGCATAGT
HSR201 I456G	CGGGATCCATGGATTCAAAGCAATCATCAG	AACTAGTCAAAGGGCAGGTCTGCCAATGGCATA
HSR201 R457G	CGGGATCCATGGATTCAAAGCAATCATCAG	AACTAGTCAAAGGGCAGGTCCGATAATGGC
HSR201 P458G	CGGGATCCATGGATTCAAAGCAATCATCAG	AACTAGTCAAAGGGCACCTCTGATAATGGC
HSR201 A459G	CGGGATCCATGGATTCAAAGCAATCATCAG	AACTAGTCAAAGGCCAGGTCTGATAATGGC
HSR201 L460G	CGGGATCCATGGATTCAAAGCAATCATCAG	AACTAGTCAACCGGCAGGTCTGATAATGGC
HSR201 I455G/I456G	CGGGATCCATGGATTCAAAGCAATCATCAG	AACTAGTCAAAGGGCAGGTCTGCCACCGGCATAGT
HSR201 I455G/A459G	CGGGATCCATGGATTCAAAGCAATCATCAG	AACTAGTCAAAGGCCAGGTCTGATACCGGCATAGT
HSR201 I456G/A459G	CGGGATCCATGGATTCAAAGCAATCATCAG	AACTAGTCAAAGGCCAGGTCTGCCAATGGCATA
Oligonucleotide pairs for the construction of HSR201 deletions fused to mVenus[a]		
HSR201(452-460)	GATCCAACTATGCCATTATCAGACCTGCCCTTTGA	CTAGTCAAAGGGCAGGTCTGATAATGGCATAGTTG
HSR201(458-460)	GATCCCCTGCCCTTTGA	CTAGTCAAAGGGCAGGG
Primer pairs for the construction of the pTV00 vectors[c]		
PEX5	GAATTCCTGCAGCCCGATGCAGCTCAAATGGCACC	ACTAGTGGATCCCCCATAACGGATGGAATCCTCAT
PEX7	GAATTCCTGCAGCCCGTGCCTTTAAGGAACATACA	ACTAGTGGATCCCCCATCACATGAGAGAATTTCAA
Primer pairs for qPCR analysis		
PEX5	GGCATGCCGTTTCGTCCTACTTCA	GGCCATGATCGTGCTACGGTCAC
PEX7	AAGATGCCGGTATTCAGAACC	ACTCTGCCGTTACCAAGGAT
Primer pairs for the construction of the pRS313 and pRS315 vectors[d]		
pRS315 with GAL1 promoter	TCTAGATAATCTCTGCTTTTGTGCG	TATAGTTTTTTCTCCTTGACGTTAAAGTATAG
pRS313 with PGK1 promoter	TCTAGATAATCTCTGCTTTTGTGCG	TGTTTTATATTTGTTGTAAAAAGTAGATAA
mVenus	GGAGAAAAAACTATAATGGTCAGTAAAGGTGAAGAGTTG	CAGAGATTATCTAGATCATTTGTACAGTTCGTCCATACCC
mVenus-HSR201	GGAGAAAAAACTATAATGGTCAGTAAAGGTGAAGAGTTG	CAGAGATTATCTAGATCAAAGGGCAGGTCTGATAATG
mCherry-PTS1	AACAAATATAAAACAATGGTATCTAAGGGCGAAGAGG	CAGAGATTATCTAGATTACAGTTTGCTTTTGTACAACTCATC
Primer pairs for the construction of the pEGFP-N1 vectors[c]		
mVenus	CGGTACCGCGGGCCCATGGTCAGTAAAGGTGAAGA	TCTAGAGTCGCGGCCTTATTTGTACAGTTCGTCCA
mVenus-HSR201	CGGTACCGCGGGCCCATGGTCAGTAAAGGTGAAGA	TCTAGAGTCGCGGCCTCAAAGGGCAGGTCTGATAA
mCherry-PTS1	CGGTACCGCGGGCCCATGGTATCTAAGGGCGAAGA	TCTAGAGTCGCGGCCTTACAGTTTGCTTTTGTACA

[a]Restriction enzyme recognition sequence and a part thereof are underlined

[b]Mutation sites are doubly underlined

[c]Nucleotide sequences of the vectors are shown in red

[d]Overlapping sequences are shown in same colors
