# Supplementary material for: Peroxisomal Localization of Benzyl Alcohol O-Benzoyltransferase HSR201 is Mediated by a Non-canonical Peroxisomal Targeting Signal and Required for Salicylic Acid Biosynthesis
Source: Plant Cell Physiol. 2024 Oct 29;65(12):2054–65. doi: 10.1093/pcp/pcae129 (PMC11662444; doi:10.1093/pcp/pcae129)
Supplement: pcae129_Supp [file pcae129_supp.zip › suppl_data/pcp-2024-e-00210-File010.pdf]

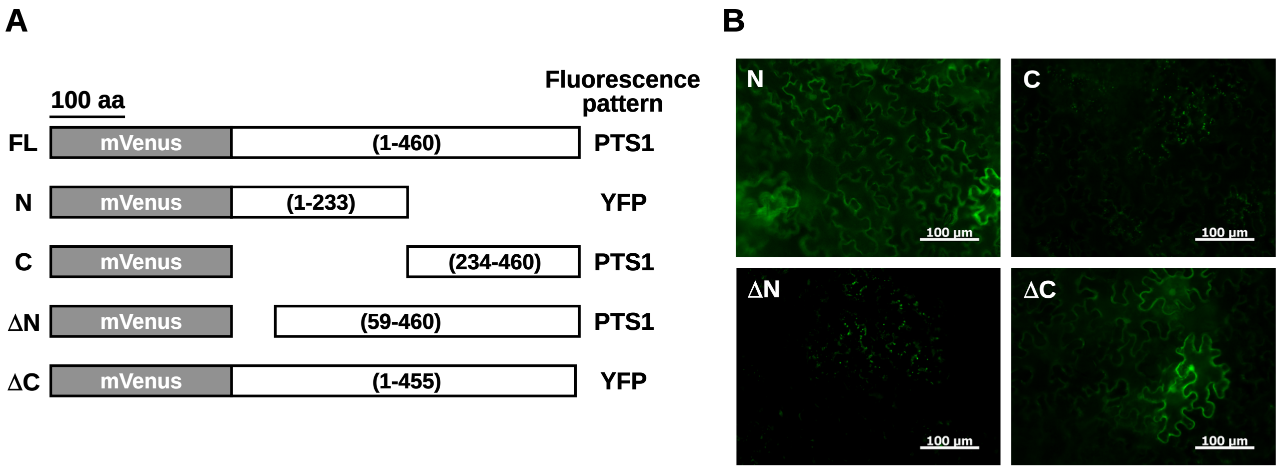

Supplementary Fig. S1 The C-terminal region of HSR201 is responsible for its peroxisomal localization. (A) A schematic representation of the deletion constructs of HSR201 fused to mVenus. The fluorescence pattern of HSR201 deletions is summarized on the right. PTS1, a fluorescent pattern similar to that of mVenus-PTS1. YFP, a fluorescent pattern similar to that of mVenus only. (B) *Agrobacterium* cells carrying any one of the indicated mVenus-HSR201 deletions expressed from a modified 35S promoter were infiltrated into *N. benthamiana* leaves. Two days after infiltration, fluorescence was observed. Bar = 100 µm.
