# Supplementary material for: Peroxisomal Localization of Benzyl Alcohol O-Benzoyltransferase HSR201 is Mediated by a Non-canonical Peroxisomal Targeting Signal and Required for Salicylic Acid Biosynthesis
Source: Plant Cell Physiol. 2024 Oct 29;65(12):2054–65. doi: 10.1093/pcp/pcae129 (PMC11662444; doi:10.1093/pcp/pcae129)
Supplement: pcae129_Supp [file pcae129_supp.zip › suppl_data/pcp-2024-e-00210-File011.pdf]

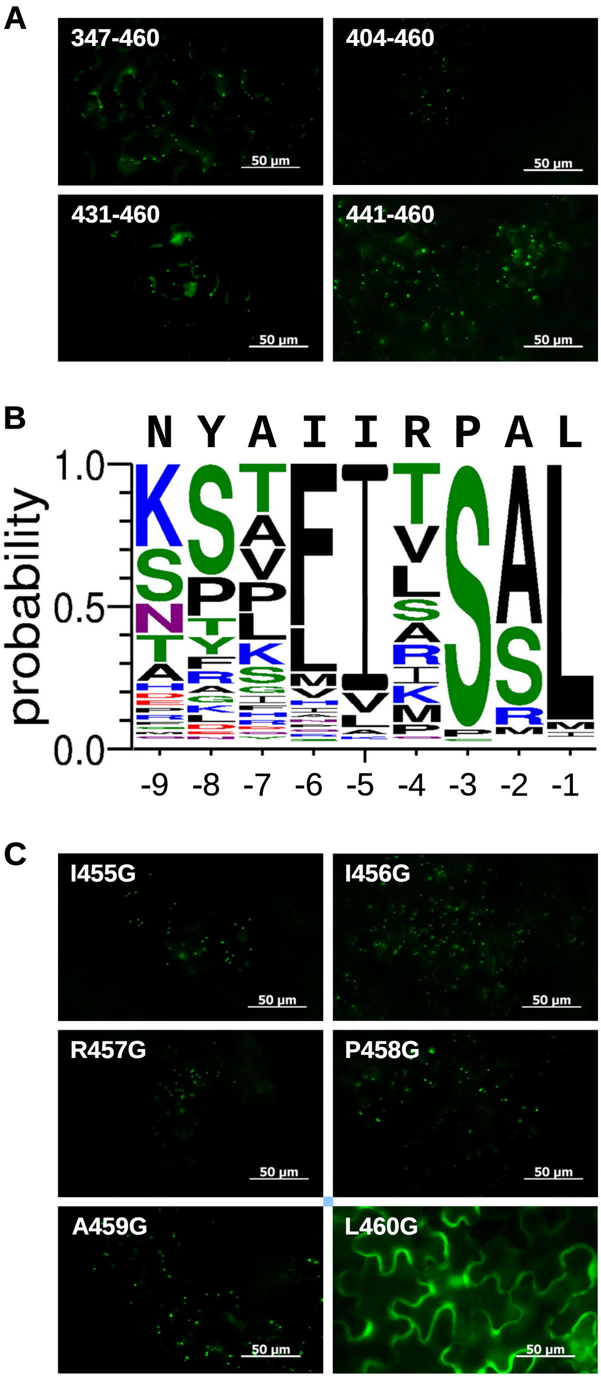

Supplementary Fig. S2 Identification of the amino acid residues of HSR201 required for its peroxisomal localization. (A) *Agrobacterium* cells carrying any one of the indicated mVenus-HSR201 deletions expressed from a modified 35S promoter were infiltrated into *N. benthamiana* leaves. Two days after infiltration, fluorescence was observed. Bar = 50 µm. (B) Web-logo image of C-termini of HSR201 homologs. The nine C-terminal amino acid sequences of HSR201 homologs were subjected to WebLogo 3 (https://weblogo.threeplusone.com). The sequence of HSR201 is shown above the logo. (C) *Agrobacterium* cells carrying any one of the indicated mVenus-HSR201 mutants expressed from a modified 35S promoter were infiltrated into *N. benthamiana* leaves. Two days after infiltration, fluorescence was observed. Bar = 50 µm.
