# Supplementary material for: Peroxisomal Localization of Benzyl Alcohol O-Benzoyltransferase HSR201 is Mediated by a Non-canonical Peroxisomal Targeting Signal and Required for Salicylic Acid Biosynthesis
Source: Plant Cell Physiol. 2024 Oct 29;65(12):2054–65. doi: 10.1093/pcp/pcae129 (PMC11662444; doi:10.1093/pcp/pcae129)
Supplement: pcae129_Supp [file pcae129_supp.zip › suppl_data/pcp-2024-e-00210-File012.pdf]

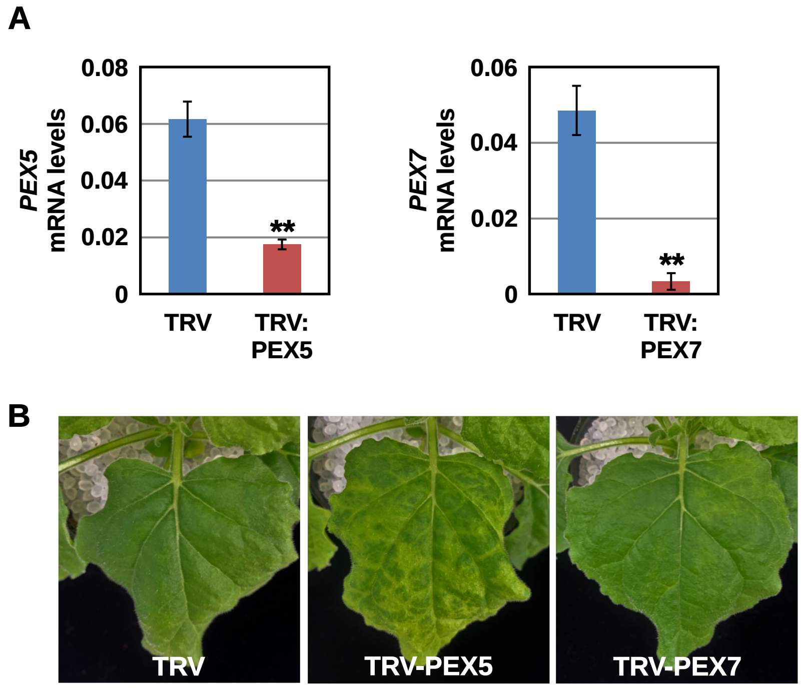

Supplementary Fig. S3 Suppression of *PEX5* and *PEX7* by virus-induced gene silencing in *N. benthamiana*. (A) *N. benthamiana* plants were infected with TRV (control), TRV:PEX5 or TRV:PEX7. Two weeks later, the transcript levels of the target genes were measured by RT-qPCR with *EF1*α as an internal standard. Values are the means with standard errors of three to four biological replicates. The significance of differences between control and silenced plants was assessed using the Student's *t*-test with Excel 2021 software (***P* < 0.01). (B) *N. benthamiana* plants were infected with TRV (control), TRV:PEX5 or TRV:PEX7 and photographed after two weeks.
