# Supplementary material for: Peroxisomal Localization of Benzyl Alcohol O-Benzoyltransferase HSR201 is Mediated by a Non-canonical Peroxisomal Targeting Signal and Required for Salicylic Acid Biosynthesis
Source: Plant Cell Physiol. 2024 Oct 29;65(12):2054–65. doi: 10.1093/pcp/pcae129 (PMC11662444; doi:10.1093/pcp/pcae129)
Supplement: pcae129_Supp [file pcae129_supp.zip › suppl_data/pcp-2024-e-00210-File013.pdf]

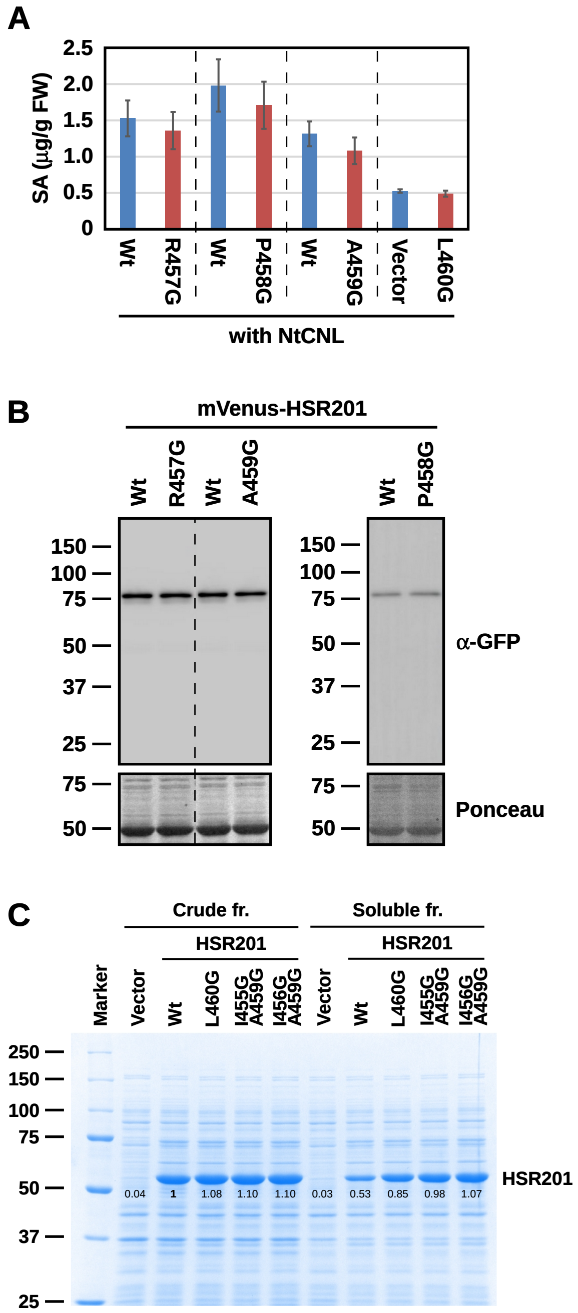

Supplementary Fig. S4 The peroxisomal localization of HSR201 is required to enhance NtCNL-induced SA biosynthesis. (A) *Agrobacterium* cells carrying *NtCNL* expressed from a modified 35S promoter were mixed with those containing any one of the indicated mVenus-HSR201 wild-type and mutants expressed from the same promoter at a ratio of 1 : 1, and the mix was then infiltrated into *N. benthamiana* leaves. *Agrobacterium* carrying an empty vector was used as a control (Vector). Two days later, SA levels were measured. Values are the means with standard errors of three to eight biological replicates. The significance of differences between the HSR201 mutants and HSR201 wild-type or the empty vector was assessed using the Student's *t*-test with Excel 2021 software, and no significant differences were detected ($P > 0.05$). (B) *Agrobacterium* cells carrying any one of the indicated mVenus-HSR201 wild-type and mutants were infiltrated into *N. benthamiana* leaves. Two days later, the accumulation of proteins was detected by immunoblotting analyses using an anti-GFP antibody (α-GFP). As a loading control, blots were stained with Ponceau-S (Ponceau). The positions of molecular mass markers in kilodaltons are indicated on the left. Experiments were repeated twice with similar results. (C) Recombinant proteins of the HSR201 wild-type (Wt), L460G, I455G/A459G and I456G/A459G were expressed in *E. coli*. Crude protein fractions (crude fr.) were prepared from cells by sonication. After the centrifugation of crude protein fractions, supernatants were used as a soluble protein fraction (soluble fr.). Crude and soluble protein fractions were separated on an SDS-polyacrylamide gel and stained with Coomassie brilliant blue. *E. coli* transformed with an empty vector were used as a control (Vector). The positions of molecular mass markers in kilodaltons are indicated on the left. Numerical values on the gel indicate the intensities of HSR201 protein bands normalized to that in the wild-type crude protein fraction, which was given a value of 1. Experiments were repeated twice with similar results.
