# Supplementary material for: Peroxisomal Localization of Benzyl Alcohol O-Benzoyltransferase HSR201 is Mediated by a Non-canonical Peroxisomal Targeting Signal and Required for Salicylic Acid Biosynthesis
Source: Plant Cell Physiol. 2024 Oct 29;65(12):2054–65. doi: 10.1093/pcp/pcae129 (PMC11662444; doi:10.1093/pcp/pcae129)
Supplement: pcae129_Supp [file pcae129_supp.zip › suppl_data/pcp-2024-e-00210-File014.pdf]

```
VIGS insert    GATACAAAGGGTACAATTATCCCCTTAGACGACATGGTTCACAAATCTTTCTTTTTTGGC
HSR201         GATACAAAGGGTACAATTATCCCCTTAGACGACATGGTTCACAAATCTTTCTTTTTTGGC
mHSR201        GACACGAAAGGAACCATCATTCCACTTGATGATATGGTACATAAGTCCTTTTTCTTCGGT
               ** ** ** ** ** ** ** **  * ** ** ***** ** ** ** ** ** ** **

VIGS insert    CCTTCTGAAGTCTCAGCACTTCGTCGATTTGTCCCTCAGCACTTGCATAAGTGTTCCACT
HSR201         CCTTCTGAAGTCTCAGCACTTCGTCGATTTGTCCCTCATCACTTGCGTAAGTGTTCCACT
mHSR201        CCAAGTGAGGTTTCTGCTTTGAGAAGGTTCGTTCCGCATCATCTGAGGAAATGCAGCACA
               **   *** ** ** **  *  *  * ** ** ** ** **  **   ** **   ***

VIGS insert    TTTGAACTTCTCACAGCAGTCCTTTGGCGTTGTCGAACAATGTCCCTAAAACCTGATCCA
HSR201         TTTGAACTGCTCACAGCAGTCCTTTGGCGTTGTCGAACAATGTCCCTAAAACCTGATCCA
mHSR201        TTCGAGCTACTTACTGCCGTTTTGTGGAGATGTAGGACTATGAGTCTCAAGCCAGACCCT
               ** ** ** ** ** ** **  * *** * *** * ** ***   ** ** ** ** **

VIGS insert    GAAGAGGAAGTTCGCGTTCTTTGCATTGTCAATGCACGTTCGAGGTTCAATCCTCCTTTG
HSR201         GAAGAGGAAGTTCGCGCTCTTTGCATTGTCAATGCACGTTCGAGGTTCAATCCTCCTTTG
mHSR201        GAGGAAGAGGTGAGAGCTTTGTGTATAGTGAACGCAAGAAGTCGATTTAACCCACCACTT
               ** ** ** **  * * * * ** ** ** ** *** *     * ** ** ** **  *

VIGS insert    CCTACTGGCTACTACGGCAATGCCTTTGCATTCCCCGTAGCAGTCACAACTGCGGCTAAA
HSR201         CCTACTGGCTACTACGGCAACGCCTTTGCATTCCCTGTAGCAGTCACAACTGCGGCTAAA
mHSR201        CCAACAGGGTATTATGGTAACGCTTTCGCGTTTCCTGTTGCTGTAACTACAGCAGCAAAG
               ** ** ** ** ** ** ** ** ** ** ** ** ** ** ** ** ** ** ** **
```

Supplementary Fig. S5 Nucleotide sequence alignment of the *NbHSR201* fragment inserted into the pTV00 vector with the corresponding regions of *HSR201* and *mHSR201*. Synonymous mutations in *mHSR201* are shown in red.
