# Supplementary material for: Peroxisomal Localization of Benzyl Alcohol O-Benzoyltransferase HSR201 is Mediated by a Non-canonical Peroxisomal Targeting Signal and Required for Salicylic Acid Biosynthesis
Source: Plant Cell Physiol. 2024 Oct 29;65(12):2054–65. doi: 10.1093/pcp/pcae129 (PMC11662444; doi:10.1093/pcp/pcae129)
Supplement: pcae129_Supp [file pcae129_supp.zip › suppl_data/pcp-2024-e-00210-File015.pdf]

```
HSR201 452-NYAIIRPAL-460
PhBPBT 452-KYAFITPAL-460
PtSABT 451-HSKFIASSL-459
PtBEBT 452-KSKFIVSSL-460
           :  :* .:*
```

Supplementary Fig. S6 Multiple sequence alignment of C-terminal amino acid sequences of HSR201, PhBPBT (AY611496), PtSABT (Potri.013G074500) and PtBEBT (Potri.019G043600). Asterisks (*), colons (:) and a period (.) indicate identical, conserved and semi-conserved amino acid residues, respectively. The amino acid residues of HSR201 that are important and essential for its peroxisomal localization are shown in blue and red, respectively.
